# Inductor coil of the highest possible $\mathbf {Q}$

**DOI:** 10.1038/s41598-020-72308-9

**Published:** 2020-09-21

**Authors:** A. Rikhter, M. M. Fogler

**Affiliations:** Department of Physics, University of California San Diego, 9500 Gilman Drive, La Jolla, CA 92093 USA

**Keywords:** Applied physics, Electronics, photonics and device physics, Electrical and electronic engineering

## Abstract

The geometry of an inductor made of a long thin wire and having the highest possible Q-factor is found by numerical optimization. As frequency increases, the Q-factor first grows linearly and then according to a square-root law, while the cross-section of the optimal coil evolves from near-circular to sickle-shaped.

## Introduction

Given a piece of wire, how can one wind it into a coil of the maximum possible *Q*-factor? While previously this question has been treated almost exclusively in the context of radio engineering^1,2^, in this work we address it as a problem in mathematical physics. To constrain the size of the coil, we have the following geometric parameters fixed: the total wire length *W*, the conducting core diameter $d_i$, and the effective outer diameter *d*. We define *d* in terms of the maximum possible wire density $n_2 \equiv (\pi d^2 / 4)^{-1}$ per unit area. Thus, for the hexagonal closed packing of round wires, *d* is $(12 / \pi ^2)^{1 / 4} = 1.050$ times the actual outer diameter. The current is taken to be $I = e^{-i\omega t}$. We consider only frequencies $\omega$ much smaller than the self-resonance frequency $\omega _r \sim c / W$ of the coil, allowing us to neglect the capacitance term. With these simplifying assumptions, the current is uniform along the wire, and the *Q*-factor is defined as the ratio of the stored magnetic energy to the magnetic losses. For the purpose of this paper, an equivalent and more convenient definition of *Q* is the ratio of the imaginary and real parts of the complex impedance $Z = R + \text {i} \omega L$:

1 $$\begin{aligned} Q(\omega ) = \frac{{{\,\mathrm{Im}\,}}Z}{{{\,\mathrm{Re}\,}}Z} = \frac{\omega L(\omega )}{R(\omega )}. \end{aligned}$$

Because of induced eddy currents, $R(\omega )$ is coil-shape dependent, so that the competition between the inductance and the losses poses a nontrivial optimization problem for $Q(\omega )$.

**Figure 1:**
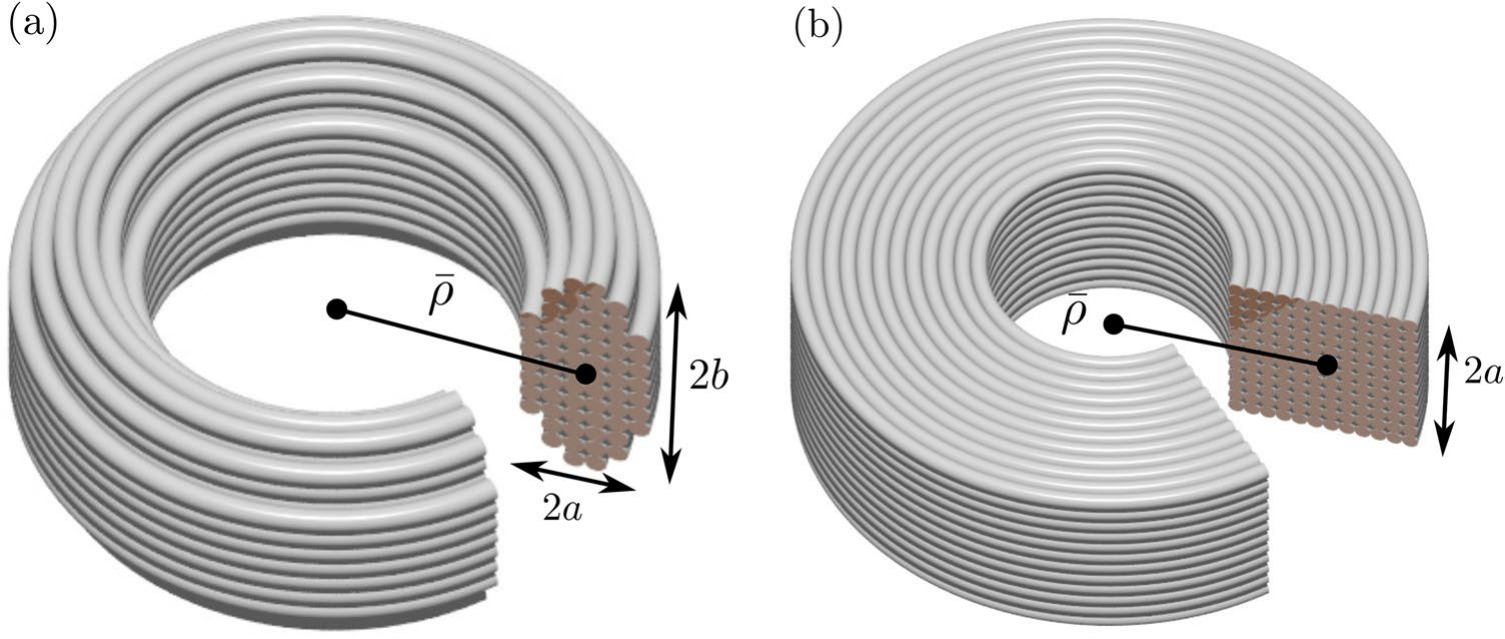
Schematics of multi-layer coils with (**a**) elliptic and (**b**) square cross-sections.

Our electrodynamic problem has roots in a magnetostatic problem first studied by Gauss^3^. Specifically, in the limit $\omega \rightarrow 0$, the effective resistance *R* approaches the dc resistance $R(0) = 4 W / (\pi \sigma d_i^2)$, where $\sigma$ is the core conductivity, so that maximizing *Q* is equivalent to maximizing *L*. Gauss assumed that the coil of the highest *L* under the aforesaid constraints is a toroidally wound solenoid with a nearly circular cross-section, Fig. 1a. Later, Maxwell^4^ revisited the problem and treated a more practical case of a square cross-section, Fig. 1b. Maxwell’s analysis was improved by Rosa and Grover^5^. Building on their work, Brooks proposed that the mean radius of the optimal coil is approximately 3/2 of the side of the square^6^. The inductance of this coil is $0.656 L_c$, where

2 $$\begin{aligned} L_c = \frac{\mu _0}{4\pi } \frac{W^{5/3}}{d^{\,2/3}}. \end{aligned}$$

Optimization of inductors with nonmagnetic cores became topical again in the 1970’s when toroidal coils (wound in the poloidal direction) were brought in a wider use in plasma physics and energy storage research. The case of a single-layer toroid was solved by Shafranov^7,8^. Multilayer coils were studied by Murgatroyd^9,10^ who found that the inductance of the optimal toroid is $0.29 L_c$. The reduction compared to the Brooks coil is presumably because the toroid generates no stray magnetic field. Murgatroyd reviewed the 5/3 power-law of  (2) and other properties of optimal inductors in his excellent summary^9^. For example, the characteristic size of such inductors is set by

3 $$\begin{aligned} \rho _c = \frac{1}{2} ({W d^2})^{1 / 3}. \end{aligned}$$

These scaling laws apply assuming the wire bundle forming the cross-section of the coil can be approximated by a continuum current distribution, which is legitimate if the number of turns *N* is large enough. For example, the relative error in the following inductance calculation due to this approximation scales as $N^{-1/2}$, as shown by Maxwell^4^. Adopting this continuum approach, below we derive scaling laws for finite-$\omega$ optimal inductors in terms of two additional characteristic scales:

4 $$\begin{aligned} \omega _c \equiv \frac{8\pi }{Q_c} \frac{d^2}{\mu _0 \sigma d_i^4}, \qquad Q_c \equiv \frac{2 \rho _c}{d_i}. \end{aligned}$$

The former is the frequency at which the eddy-current losses become comparable with the dc Ohmic ones, the latter is the order of magnitude of the *Q*-factor at $\omega _c$.

## Low frequencies

**Figure 2:**
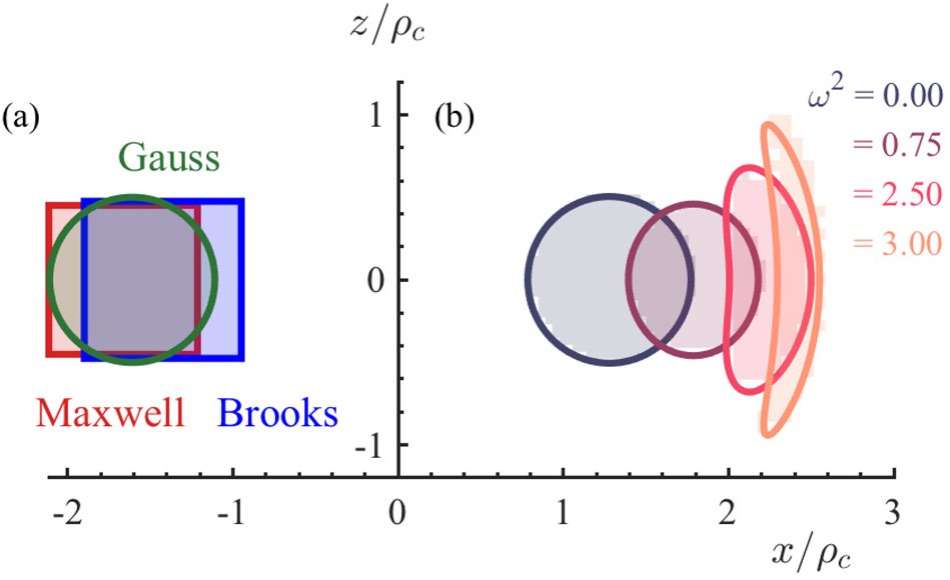
Cross-sections of the optimal coils. (**a**) Designs proposed by Gauss^3^, Maxwell^4^, and Brooks^6^. (**b**) Results obtained in this work. The cross-section evolves from near-circular to elliptic to sickle-shaped as $\omega$ increases. The shading represent the local wire density $n({\mathbf {r}})$ computed on a $30\times 30$ grid. The curves serve as guides to the eye. The wire density is seen to switch from 0 to $n_2$ with few or no intermediate values. The numbers on the axes are *x* and *z* coordinates in units of $\rho _c$. The legend indicates the magnitudes of $(\omega / \omega _c)^2$.

We begin with answering Gauss’ question about the dc inductance. It was posed by him 150 years ago but apparently has not been settled yet. Gauss’ calculation can be summarized as follows. An estimate of *L* is provided by the approximate formula^4^

5 $$\begin{aligned} L = \mu _0 N^2 {\bar{\rho }} \left[ \ln \left( \frac{8 {\bar{\rho }}}{{\mathrm {\scriptstyle GMD}}}\right) - 2\right] , \end{aligned}$$

where *N* is the total number of turns in the coil and ${\bar{\rho }}$ is their mean radius. Note that $2\pi {\bar{\rho }} N = W$. Parameter ${\mathrm {\scriptstyle GMD}}$ is the geometric mean distance. In the continuum limit, appropriate for large *N*, it is defined via

6 $$\begin{aligned} \ln ({\mathrm {\scriptstyle GMD}}) = \frac{1}{A^2} \iint \, \ln |{\mathbf {r}} - {\mathbf {r}}'| {d^2 r d^2 r'}, \end{aligned}$$

where positions ${\mathbf {r}} = (\rho , z)$, ${\mathbf {r}}' = (\rho ', z')$ vary over the cross-section of the coil, of the area $A = N / n_2$. According to  (5), to maximize *L* for a given *N* (or ${\bar{\rho }}$) we need to minimize ${\mathrm {\scriptstyle GMD}}$ at fixed *A*. It can be proven^11^ that the solution is a circle of radius $a = \sqrt{A / \pi }$ whose ${\mathrm {\scriptstyle GMD}}$ is^5^ $\text {e}^{-1 / 4} a$. Minimizing *L* with respect to ${\bar{\rho }} / a$, Gauss obtained ${\bar{\rho }} / a = \text {e}^{13 / 4} / 8 = 3.22$. Such a mean-radius to half-height ratio is noticeably different from either 3.7 or 3 advocated by, respectively, Maxwell and Brooks, see Fig. 2a, suggesting that this method is too crude to reveal the true optimal coil geometry.

To glean a more accurate answer, we tackled the problem numerically. We expressed the inductance and the wire-length constraint in the form of integrals,

7 $$\begin{aligned} L&= \iint \,n({\mathbf {r}}) M({\mathbf {r}}, {\mathbf {r}}') n({\mathbf {r}}')\, d^2r\, d^2r', \end{aligned}$$

8 $$\begin{aligned} W&= \int \,n({\mathbf {r}})\, 2\pi \rho \, d^2r, \end{aligned}$$

where $0 \le n({\mathbf {r}}) \le n_2$ is the number of turns per unit area at position ${\mathbf {r}}$. Function $M({\mathbf {r}}, {\mathbf {r}}')$, given by

9 $$\begin{aligned} \begin{aligned} M({\mathbf {r}}, {\mathbf {r}}')&= \mu _0 \sqrt{\frac{\rho \rho '}{m}} \left[ (2 - m) K(m) - 2 E(m)\right] , \\ m&= \frac{1}{1 + k^2},\quad k = \frac{|{\mathbf {r}} - {\mathbf {r}}'|}{\sqrt{4 \rho \rho '}} \end{aligned} \end{aligned}$$

is the mutual inductance of co-axial line currents^5^ piercing the cross-section at ${\mathbf {r}}$ and ${\mathbf {r}}'$; *K*(*m*) and *E*(*m*) are the complete elliptic integrals. We approximated the integrals in  (7), (8) by sums over a finite two-dimensional grid and performed the constrained maximization of *L* numerically. The mean radius of the optimal coil is ${\bar{\rho }} = 1.28 \rho _c$. The cross-section of the coil is not a circle; it is better approximated by an ellipse of dimensions

10 $$\begin{aligned} \xi _1 \equiv \frac{\bar{\rho }}{a} = 2.54, \qquad \xi _2 \equiv \frac{\bar{\rho }}{b} = 2.61, \end{aligned}$$

represented by the curve labeled $\omega ^2 = 0$ in Fig. 2b. The cross-section is fully packed, so that

11 $$\begin{aligned} n({\mathbf {r}}) = n_2\, \Theta \left( 1 - \frac{(\rho - \bar{\rho })^2}{a^2} - \frac{z^2}{b^2}\right) , \end{aligned}$$

where $\Theta (x)$ is the unit step-function. Finally, the coil inductance is

12 $$\begin{aligned} L = 0.663 L_c, \end{aligned}$$

which is $1\%$ larger than that of the Brooks coil.

Encouraged by the simplicity of these results, we rederived them as follows. We started with the expansion^5^

13 $$\begin{aligned} M({\mathbf {r}}, {\mathbf {r}}') \simeq \mu _0\sqrt{\rho \rho '} \left[ \left( 1 + \frac{3 k^2}{4} \right) \ln \frac{4}{k} -2 - \frac{3 k^2}{4} \right] , \end{aligned}$$

valid for $k \ll 1$ [Eq. (9)], and evaluated the integral in  (7) analytically for the elliptic cross-section defined by  (11). The result can be written as

14 $$\begin{aligned} L&= \mu _0 N^2 \bar{\rho }\, \Lambda , \end{aligned}$$

15 $$\begin{aligned} \Lambda&= \left( 1 + \frac{1}{32} \frac{\xi _2^{2} + 3 \xi _1^{2}}{\xi _1^{2} \xi _2^{2}}\right) \ln \left( \frac{16\, \xi _1 \xi _2}{\xi _1 + \xi _2}\right) - \frac{7}{4} \nonumber \\&\quad + \frac{7}{96} \frac{1}{\xi _1^2} + \frac{1}{32} \frac{\xi _2^{2} - 3 \xi _1^{2}}{\xi _1^{2} \xi _2^{2}} \frac{\xi _1}{\xi _1 + \xi _2}, \end{aligned}$$

which is a generalization of Rayleigh’s formula^12^ for the $b = a$ case and a key improvement over  (5). Using this formula for *L* and another one, $W = \pi a b \bar{\rho } n_2$, for the length constraint, we were able to easily solve for the optimal $\xi _1$, $\xi _2$ numerically, reproducing  (10).

Returning to the *Q*-factor, we rewrite  (1) in terms of our characteristic scales $L_c$, $Q_c$, $\omega _c$:

16 $$\begin{aligned} Q = \frac{\pi }{2} \frac{\omega }{\omega _c} \frac{L / L_c}{1 + F(\omega )} Q_c, \end{aligned}$$

where we introduced the loss enhancement factor

17 $$\begin{aligned} F(\omega ) \equiv \frac{R(\omega )}{R(0)} - 1. \end{aligned}$$

Below we show that at low frequencies $\omega \ll \omega _c$, the loss factor behaves as

18 $$\begin{aligned} F(\omega ) = 0.305\, \frac{\omega ^2}{\omega _c^2}. \end{aligned}$$

At such frequencies, $F \ll 1$ is negligible, *L* is virtually unchanged from the dc value, and so the *Q*-factor is linear in $\omega$:

19 $$\begin{aligned} \frac{Q}{Q_c} = 1.04\, \frac{\omega }{\omega _c}, \quad \omega \ll \omega _c, \end{aligned}$$

see Fig. 3.

## Proximity effect losses

The finite-frequency losses in coils are traditionally attributed to the combination of the skin and proximity effects^13^. The latter, due to the collective field ${\mathbf {H}}({\mathbf {r}})$ of all the turns of the wire, dominates in multi-layer coils of interest to us if $\omega$ is not too high, such that $\delta \gg d_i$, where

20 $$\begin{aligned} \delta (\omega ) = \sqrt{\frac{2}{\mu _0 \omega \sigma }} \end{aligned}$$

is the skin depth. Under the stated condition of weak skin effect, the loss factor takes the form

21 $$\begin{aligned} F(\omega ) = \frac{\pi ^2}{64 W} \frac{d_i^6}{\delta ^4} \int \,{\mathbf {H}}^2({\mathbf {r}}) n({\mathbf {r}})\, 2\pi \rho \, d^2r \end{aligned}$$

where ${\mathbf {H}}$, equal to the curl of a vector potential, is

22 $$\begin{aligned} {\mathbf {H}}({\mathbf {r}}) = \frac{1}{2\pi \mu _0\rho } (\hat{{\mathbf {z}}} \partial _\rho - \hat{{\varvec{\rho }}} \partial _z) \int \,M({\mathbf {r}}, {\mathbf {r}}') n({\mathbf {r}}') \text {d}^2r'. \end{aligned}$$

In general, these expressions have to be evaluated numerically. However, we can estimate *F* analytically for a coil with the elliptic cross-section,  (11). Retaining only the leading-order terms in $k \sim \max (a, b) / \bar{\rho } \ll 1$ in  (13), we find

23 $$\begin{aligned} {\mathbf {H}}({\mathbf {r}}) = \frac{n_2}{a + b} \left[ a z\, \hat{{\varvec{\rho }}} - (\rho - \bar{\rho }) b\, \hat{{\mathbf {z}}} \right] . \end{aligned}$$

Substituting this into  (21), we get

24 $$\begin{aligned} F = \frac{1}{8} \left( \frac{d_i^3}{\delta ^2 d^2} \frac{a b}{a + b}\right) ^2 = \frac{\pi ^2}{2}\, \frac{\omega ^2}{\omega _c^2} \left( \frac{1}{\rho _c}\, \frac{a b}{a + b}\right) ^2, \end{aligned}$$

which is a generalization of Howe’s formula for a multi-stranded round wire^14^. Finally, using  (3) and (10), we arrive at  (18). At the border of its validity, $\omega \approx \omega _c$, that equation predicts $F \approx 0.3$ assuming the wire is long enough so that $\delta / d_i \approx 0.2\, (W/d)^{1/6} \gg 1$.

**Figure 3:**
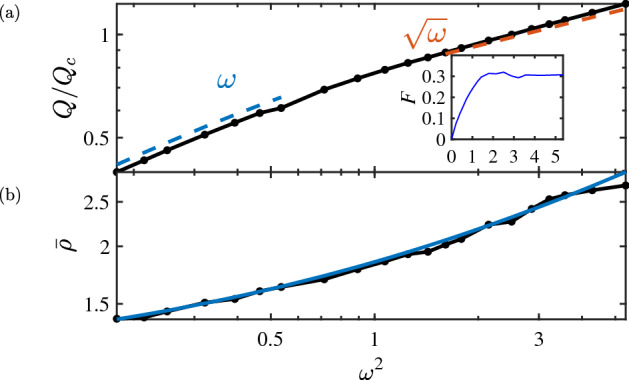
(**a**) *Q*-factor of the optimal coil as a function of $\omega ^2 / \omega _c^2$. The connected dots are our numerical results. The two dashed lines indicate the expected low- and intermediate-frequency scaling. Inset: loss factor *F* *vs*. $\omega ^2 / \omega _c^2$. (**b**) Mean radius $\bar{\rho }$ of the coil in units of $\rho _c$ as a function of $\omega ^2 / \omega _c^2$.

## Intermediate frequencies

At $\omega \gg \omega _c$ the competition between inductance and proximity losses is expected to cause flattening of the cross-section of the optimal coil. We confirmed this hypothesis by numerical simulations based on  (7), (8), (21), and (22). Our results for a few representative $\omega$ are shown in Fig. 2b. As frequency increases, the cross-section first becomes oval and then sickle-shaped. Figure 3 presents the *Q*-factor and the mean radius $\bar{\rho }$ obtained from these simulations. The plot in the main panel of Fig. 3a suggests that the linear scaling of $Q(\omega )$ changes to a square-root law above the frequency $\omega _c$ as the cross-section begins to flatten and bend. The inset of Fig. 3a illustrates that the loss factor grows as predicted by  (18) at $\omega / \omega _c < 1$ but reaches a constant $F \approx 0.3$ at $\omega / \omega _c > 1$.

We can shed light on the observed $\omega / \omega _c > 1$ behaviors using our elliptical cross-section model. Assuming $a\ll b$, we derive the following analytical expressions for *a* and *b* in terms of dimensionless parameters $\xi _2 = \bar{\rho } / b$ and *F*:

25 $$\begin{aligned} \frac{a}{\rho _c} = \sqrt{\frac{F}{2\pi ^2}}\, \frac{\omega _c}{\omega }\,, \qquad \frac{b}{\rho _c} = \sqrt{\frac{1}{\pi \xi _2} \frac{\rho _c}{a}}. \end{aligned}$$

They entail that *Q* at a given $\omega$ has the scaling form

26 $$\begin{aligned} Q(\xi _2, F) = \frac{F^{1 / 4}}{1 + F}\, q\left( \xi _2\right) . \end{aligned}$$

Hence, *Q* at fixed $\xi _2$ reaches its maximum at $F = 1/3$, which is close to our numerical result. Freezing *F* at 1/3 and maximizing *Q* with respect to $\xi _2$, we arrived at

27 $$\begin{aligned} \frac{Q}{Q_c}&= 0.85\, \sqrt{\frac{\omega }{\omega _c}}\,,&\frac{\bar{\rho }}{\rho _c}&= 1.6\, \sqrt{\frac{\omega }{\omega _c}}\,, \end{aligned}$$

28 $$\begin{aligned} \frac{a}{\rho _c}&= 0.26\, \frac{\omega _c}{\omega },&\xi _2&= 2.13. \end{aligned}$$

The first equation in  (27), represented by the upper dashed line in Fig. 3a, is within $10\%$ from the simulation results. The second equation in  (27), has a similar level of agreement with the data in Fig. 3b. This is satisfactory considering that $\omega / \omega _c$ is not truly large and that our analytical model is oversimplified.

## High frequencies

From now on we focus on the practical case of densely packed, thinly insulated wires, $d_i \approx d$. Per  (20) and (25), at frequency $\omega _s = \omega _c Q_c / (2\pi ) \gg \omega _c$ both the width 2*a* of the thickest part of the winding and the skin depth $\delta$ become of the order of *d*. This implies that at $\omega \gg \omega _s$ the optimal coil is (i) single-layered and (ii) strongly affected by the skin effect. In view of the former, we can fully specify the cross-sectional shape of the coil by a function $\rho (z)$ and replace  (7) and (8) by

29 $$\begin{aligned} L&= n_1^2 \iint \, M({\mathbf {r}}, {\mathbf {r}}') ds\, ds', \end{aligned}$$

30 $$\begin{aligned} W&= n_1 \int \,2\pi \rho (z) ds, \quad \text {d} s = \sqrt{1 + \rho ^{\prime 2}(z)}\, dz, \end{aligned}$$

with $n_1 \sim 1 / d$ being the number of turns per unit arc length *s* of the cross-section. Equation (21) gets modified as well. As first shown by Rayleigh^15^, a single straight round wire is characterized by the loss factor $F_s = {d_i} / ({4\delta }) \gg 1$, due to confinement of the current to a $\delta$-thick skin layer at the conductor surface. In a coil or in a bunch of parallel wires, inter-wire interactions cause further nonuniformity of the current in the skin layer. As a result, the loss factor increases beyond Rayleigh’s $F_s$:

31 $$\begin{aligned} \frac{F}{F_s} = \lambda + \frac{d_i^2 n_1}{8 W} \int \, \left[ f {H}^2_{\parallel }(z) + g {H}^2_{\perp }(z)\right] 2\pi \rho \, ds, \end{aligned}$$

where ${H}_{\parallel }(z)$ and ${H}_{\perp }(z)$ are the components of ${\mathbf {H}}({\mathbf {r}})$ parallel and perpendicular to the layer,

**Figure 4:**
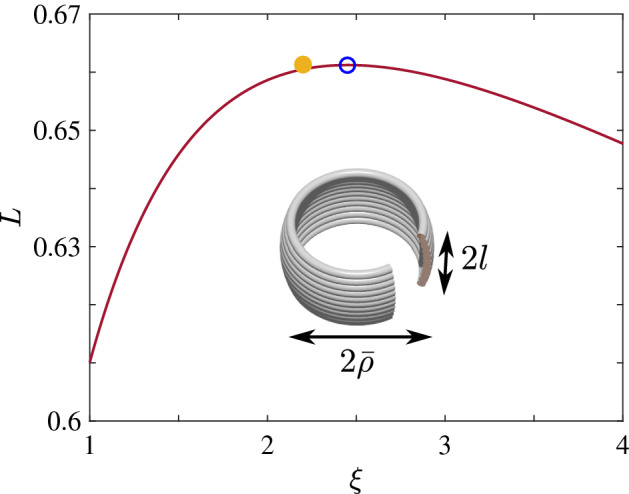
Inductance *L* of a constant-radius single-layer coil as a function of $\xi = \bar{\rho } / l$. The open dot labels the maximum on the curve. The filled dot shows the true optimum. *L* is in units of $\mu _0 W^{3 / 2} / (2\pi \sqrt{d})$. Inset: definitions of $\bar{\rho }$ and *l*.

32 $$\begin{aligned} {H}_{\parallel }(z) = \frac{H_\rho \rho ' + H_z}{\sqrt{1 + \rho ^{\prime 2}}}, \qquad {H}_{\perp }(z) = \frac{H_\rho - H_z \rho '}{\sqrt{1 + \rho ^{\prime 2}}}. \end{aligned}$$

The dimensionless coefficients $\lambda$, *f*, and *g* introduced by Butterworth^13^ depend on the wire packing density $n_1 d_i$ and have to be calculated numerically^16^. The optimization of *Q* using the entire set of these complicated equations appears to be challenging, so we have not attempted it. On the other hand, the solution for $\rho (z)$ we present below is a nearly constant function. For such functions the loss factor *F* should be weakly shape dependent, in which case to maximize *Q* it is sufficient to maximize *L* alone. We accomplished the latter numerically using  (29) and (30), in which we additionally dropped the $\sqrt{1 + \rho ^{\prime 2}}$ factors. The optimal solenoid shape we found is slightly convex, as depicted schematically in Fig. 4, with the aspect ratio $\xi = \bar{\rho } / l = 2.20$ and curvature 0.0024/*l*. Note that $\xi$ is numerically close to $\xi _2$ in the intermediate frequency regime,  (28). Substituting the obtained *L* into  (16), we got

33 $$\begin{aligned} \frac{Q}{Q_c} = \frac{2.34}{F / F_s}\, \sqrt{\frac{\omega }{\omega _c}}\,, \quad \omega \gg \omega _s, \end{aligned}$$

which is similar to  (27) but has a different coefficient. This type of high-frequency behavior is actually well-known in radio engineering^1,2^.

In an effort to rederive these results more simply, we considered a family of constant-radius solenoids whose inductance is given by Lorenz’s formula^5^

34 $$\begin{aligned} L = \frac{8}{3} \mu _0 n_1^2 \rho ^3 \left[ \frac{2m - 1}{m \sqrt{m}} E(m) + \frac{1 - m}{m \sqrt{m}} K(m) - 1\right] , \end{aligned}$$

where $m = {\rho ^2} / ({\rho ^2 + l^2})$. As seen in Fig. 4, the maximization of this *L* (under the constraint $4\pi \rho l n_1 = W$) gives $L = 0.661 \mu _0 W^{3 / 2} / (2\pi \sqrt{d}\,)$, in agreement with Murgatroyd^9^. This value of the inductance is only $\sim 1\%$ lower than the true optimum, which corresponds to the slightly convex shape we found here. Yet the best aspect ratio for the constant-radius solenoid proves to be 2.46, a $13\%$ larger than for our optimal coil.

## Discussion

In this work we studied theoretically the highest possible *Q*-factor of an inductor wound from a given piece of wire. Real inductors used in various practical applications^17–20^ are made under numerous additional constraints, such as minimal cost, ease of manufacturing, or current handling capacity. Depending on the application, a multitude of related optimization problems may arise. Our calculation provides a fundamental upper bound on *Q* and its scaling with wire length, diameter, and frequency. At the highest frequencies we considered, $Q(\omega )$ grows according to the square-root law. We expect this law to persist until either capacitance effects or radiative losses or frequency dispersion of $\sigma$ neglected in our theory become important. For example, the capacitance effects restrict the validity of . (33) to frequencies below the self-resonance frequency $\omega _r \sim c / W$. Hence, this equation may apply only if $\omega _r / \omega _s \sim Z_0 / R(0) \gg 1$, i.e., if the dc resistance of the wire is much smaller than the impedance of free space $Z_0 = c \mu _0 = 377\,\Omega$. However, if *R*(0) is too low, then dipole radiation^21,22^ losses, growing as $\omega ^4$, could surpass the Ohmic ones. Consideration of these additional physical effects is relevant for optimizing inductors used in resonators, antennas, and metamaterials, and so it could be an interesting topic for future research.

## Methods

To optimize inductance, the Eqs. (7) and (8) were replaced by sums:

35 $$\begin{aligned}&L[n] = \sum _i \sum _jn_i M_{ij} n_j \end{aligned}$$

36 $$\begin{aligned}&W[n] = \sum _i2\pi \rho _in_i \end{aligned}$$

over finite two-dimensional grid of points ${\mathbf {r}}_i$ in the cross-section of the coil . The off-diagonal elements $M_{ij} = M({\mathbf {r}}_i, {\mathbf {r}}_j)$ were found from (9). To find the diagonal elements of the matrix, the mutual inductance of two rings of radius $\rho _i$ offset by a vertical distance, $M({\mathbf{r}}_{{\mathbf{i}}} ,{\mathbf{r}}_{{\mathbf{i}}}  + a_{{{\text{GMD}}}} {\hat{\mathbf{z}}}))$, was computed, which is a good approximation to the self-inductance of a thin wire^23^. The matrix *M* is positive definite, and so the maximization of *L* is a convex constrained optimization problem, which we solved using MATLAB’s^24^ built-in quadprog function, yielding the optimal distribution of currents $n_i$.

The full problem including the proximity and skin effects is more complicated; to begin with, it is no longer obviously convex due to the additional factor in (21). The magnetic field entering this equation can in principle be found from (22). We used an equivalent method, as follows. For each point on a two-dimensional grid, the magnetic field vector at coordinate *i* due to the current at coordinate *j* was calculated using the well-known formula^25^ for the magnetic field of a ring current, yielding the matrix $H_{ij}$. The loss factor was then calculated using the discretized version of (21),

37 $$\begin{aligned} F[n] = \frac{\pi ^3}{32 W} \frac{d_i^6}{\delta ^4} \sum _j\left( \sum _iH_{ij} n_i\right) ^2\rho _j. \end{aligned}$$

The optimal distribution of current $n_i$ was then obtained by maximizing

38 $$\begin{aligned} Q[n] = \frac{L[n]}{1 + F[n]}, \end{aligned}$$

numerically, using the built-in fmincon function in MATLAB. This function requires an initial guess, which we chose to be random. We verified that the results of the optimization were independent of the starting values of $n_i$ and satisfied the constraint (36) up to the specified tolerance of $10^{-6}$. For guiding the eye, the values $n_i$ depicted in Fig. 2 were supplemented with smooth envelope curves. For the two lower frequencies, ellipses were used, and for the higher two, sickle-shaped curves satisfying $c_0 = (x^2 + y^2 - c_1)^2 + c_2 y^2$ were used with suitable choices of $c_0, c_1, c_2$.
